# Adipose-derived stem cell exosomes promote tumor characterization and immunosuppressive microenvironment in breast cancer

**DOI:** 10.1007/s00262-023-03584-3

**Published:** 2024-01-31

**Authors:** Qin Zhu, Yukun Cao, Jiaqi Yuan, Yu Hu

**Affiliations:** Department of General Surgery, Xiangya Hospital, Central South University, No. 87 Xiangya Road, Changsha, 410008 Hunan People’s Republic of China

**Keywords:** Adipose-stem cell, Exosomes, Breast cancer, T cell, Tumor microenvironment

## Abstract

Adipose-derived stem cells (ASC) or autologous fat transplantation could be used to ameliorate breast cancer postoperative deformities. This study aims to explore the action of ASC and ASC-exosomes (ASC-exos) in breast cancer characterization and tumor microenvironment immunity, which provided a new method into the application of ASC-exos. ASC were extracted from human adipose tissue for the isolation and verification of ASC-exos. ASC-exos were co-cultured with CD4^+^T cells, CD14+ monocytes and MCF-7 cells, respectively. The tumor formation of nude mice was also constructed. Cell characterization was determined by CCK8, scratch assay, and Transwell. Hematoxylin–eosin (HE), immunohistochemistry (IHC) and immunofluorescence (IF) staining were used to observe the histopathology and protein expression. CD4^+^T cell and CD14+ monocytes differentiation was detected by flow cytometry. Western blot, qRT-PCR and RNAseq were used to detect the action of ASC-exos on gene and protein expression. CD4^+^T cells could take up ASC-exos. ASC-exos inhibited Th1 and Th17 differentiation and promoted Treg differentiation of CD4^+^T cells. ASC-exos inhibited M1 differentiation and promoted M2 differentiation of CD14+ monocytes. ASC-exos promoted the migration, proliferation, and invasion, while inhibited apoptosis of MCF-7 cells. ASC-exos promoted the tumor formation of breast cancer. The effect of ASC-exos on tumor microenvironment immunity was in accordance with the above in vitro results. TOX, CD4 and LYZ1 genes were upregulated, while Mettl7b and Serpinb2 genes were downregulated in ASC-exos group. Human T-cell leukemia virus 1 infection pathway was significantly enriched in ASC-exos. Thus, ASC-exos promoted breast cancer characterization and tumor microenvironment immunosuppression by regulating macrophage and T cell differentiation.

**Supplementary Information:**

The online version contains supplementary material available at 10.1007/s00262-023-03584-3.

## Introduction

Breast cancer is the most common malignancy worldwide and the leading cause of cancer death [1]. Tumor-infiltrating lymphocytes have emerged as a clinically relevant and highly repeatable biomarker that can influence breast cancer prognosis and therapeutic response [2]. Obesity is known to be linked to a higher risk of more aggressive breast cancer and reduced survival rates for patients [3, 4]. Primary human breast cancer affects the progression of breast tumors by invading surrounding fat and contacting adipose cells, inflammatory infiltration, and fibrous interstitium [5]. In vivo, deletion of the neutrophil specific gene ATGL or ATGL inhibitor altered neutrophil lipid profiles and lung metastasis of breast tumors in mice [6]. Therefore, we suspected that adipose-derived stem cell (ASC) may be mediators of malignant characterization in breast cancer, but their specific role remains unclear.

ASC or autologous fat transplantation could be used to improve breast tissue regeneration after hysterectomy or postoperative breast cancer deformities [7, 8]. To date, the potential for ASC to promote breast cancer growth and invasion found in basic science studies has indeed not been confirmed in clinical trials [9]. At present, some studies supported the effectiveness and safety of enriching ASC from the matrix vessels of adult adipose tissue [8, 9]. However, it was not known whether transplanted or resident ASCs increased the recurrence of cancer. As a key component of breast stroma, ASC played an important role in the breast cancer microenvironment [10]. Cross-talk between ASC and breast cancer cells were multilateral, and could occur either directly through intercellular contact or indirectly through secretory bodies released by ASC/MSC, which were considered to be major effectors of their supporting, angiogenic, and immunomodulatory functions [11]. Therefore, ASC derived exosomes (ASC-exos) was intermediates took part in the malignant phenotype of breast cancer, but their role remains unknown.

ASC-exos were known to be important components of ASC paracrine release and have a variety of biological activities [12]. ASC-mediated immune/inflammatory processes in the tumor microenvironment by releasing paracrine signaling factors alone or as a cellular extracellular vesicles (EVs, exosomes) [13]. Adipose MSCs inhibited the differentiation and proliferation of T cells and reduced the production of proinflammatory cytokine interferon *γ* (INF-*γ*) [14]. MSCs exosomes could also promote M2-type macrophages to secrete the anti-inflammatory cytokines [15]. In breast cancer models, there is a lack of systematic research on the role of ASC-exos in tumor cell differentiation of T cells and macrophages as well as the regulation of Th1/Treg balance. Therefore, this study systematically studied the effects of ASC and ASC-exos on T cells, macrophages, and breast cancer cells. The potential mechanism of ASC-exos on tumor growth was analyzed by RNAseq, which may add new insights to the application of ASC-exos.

## Materials and methods

### Extraction of ASC and identification of adipogenic differentiation

Human adipose tissue was provided by the Xiangya Hospital, Central South University for the extraction of ASC. In short, the adipose tissue was cut to paste and washed. The rinsed tissue was placed in a plate and added with collagenase (0.3 μg/mL, 0.075% type II collagenase). The tissues were then transferred to a flask with a pipette and placed in a 37 °C-water bath for 30 min, with oscillations every 5–10 min. After 30 min, the digestion of collagenase was terminated by normal saline. After balance, supernatant and undigested fat were removed by centrifugation. DMEM containing fetal bovine serum (10%, FBS) was used to precipitate the suspended cells. The remaining red blood cells were dissolved with 0.16 mol/L ammonia chloride. The cell suspensions were filtered through 200 mesh copper mesh and centrifuged for 10 min (1200 r/min) to obtain mononuclear cells. The cells were added into the culture medium for count. Cell concentration was adjusted to 10^4^ cells/mL based on the count results. Adipogenic and osteogenic differentiation ability of adipose-derived stem cell (ASC) could be identified by oil red and alizarin red staining after 12 days of culture. The expression of the CD73, CD90, CD44, CD34, and HLA-DR markers in ASC was detected by flow cytometry.

### Isolation and identification of exosomes from ASC

The third generation of human ASCs were transferred to a centrifuge tube and centrifuged at 3000 g at 4 °C for 10 min to remove cell debris from the cell supernatant. To remove impurities, exosome concentration solution (ECS, UR52121, Umibio) was added to the centrifuge supernatant. For every 20 mL of sample, 5 mL of ECS reagent was added. The samples were mixed using a vortex oscillator and then placed at 2–8 °C for 2 h.

Afterward, the centrifuge tube containing the mixed liquid was taken out and centrifuged at 10,000 g at 4 °C for 60 min. The supernatant was discarded, and the precipitation was found to contain abundant exosome particles. To wash the centrifugal precipitate, 100 µL of 1 × PBS was taken and evenly sprayed onto it. The suspension was then transferred to a 1.5-mL centrifuge tube and centrifuged at 4 °C at 12,000 g. This process helped to obtain the supernatant enriched with exosome particles.

The coarse exosome particles were carefully transferred into the upper chamber of the exosome purification filter (EPF column) and centrifuged at 3000 g at 4 °C for 10 min. After centrifugation, the liquid at the bottom of the EPF column (purified exosome particles) was collected. Finally, the exosomes were characterized using electron microscopy and particle size analysis. The expressions of CD9, CD63, and CD81, Tubulin and β-actin in the exosomes were tested using western blot.

### Transmission electron microscopic observation of ASC-exo

The exosomes were resuspended in 100 μL of 2% PFA (P6148, SIGMA). Then, 5 μL of the exosome suspension was added to a formvar-carbon sample carrier copper mesh (01753-F, PELCO). Subsequently, 100 μL of PBS was added to the sealing film, and the copper mesh (with the Formvar membrane facing down) was carefully placed on the PBS droplets using tweezers for cleaning purposes. Afterward, the copper mesh was placed in a 50-μL droplet of 1% glutaraldehyde (16,051, Ted-pella) for 5 min. Following that, the copper mesh was cleaned by placing it in 100 μL of ddH_2_O_2_ (E130-01A, Novoprotein) for 1 min. It was then transferred to a 50-μL droplet of uranium oxalate dioxyoxide liquid for 5 min. After cleaning, the copper mesh was placed on 50 droplets of methyl cellulose for 10 min while kept on ice. Next, the copper mesh was positioned in a stain-less steel ring, and any excess liquid was absorbed and allowed to dry. Finally, the copper mesh was placed in a box and imaged under an electron microscope (1230, JEOL) at 80 kV.

### Exosomes uptake test

Exosomes were resuspended with 1 ml PBS. PKH67 reagent (2 µL) was added and incubated at 37 °C for 20 min to label exosomes. Serum (10 µL) was added to terminate the reaction. Labeled exosomes (100 µL) were added to the climbing sheets to cover the cells. Then, cells climbing sheets were incubated at 37 °C for 30 min–1 h and washed with PBS. DAPI (1 mg/mL) was added to cells climbing sheets and incubated, which were observed and photographed under fluorescence microscope (BA210T, Motic).

### Cell experiment and grouping

The ASC-exos [16, 17] were divided into Control group, 1 μg/mL group, 5 μg/mL group, and 10 μg/mL group. ASC-exos were co-culture with 2 × 10^5^ CD4^+^T cell for 6 days to analyze the differentiation [14]. ASC-exos were co-culture with 2 × 10^5^ CD14 + monocytes for 30 h to analyze the M1/M2 phenotype [18]. ASC-exos were co-culture with 5 × 10^6^ MCF-7 cells (AW-CCH139, Abiowell) for 2 days to detect cell characterization.

The separation of CD14 + monocytes from human peripheral blood was performed as follows. LymphoPrep (#07811, Axis-Shield) was used to purify cells by density gradient centrifugation. CD14 MicroBeads (human, 130-050-201, Miltenyi) was applied to sort CD14 + monocytes. CD14 + monocytes were cultured in RPMI 1640 medium.

CD4^+^T cells were sorted by kit. The separation of CD4^+^T cells from human peripheral blood was performed by EasySep™ Human CD4^+^T Cell Isolation Kit (17,952, STEMCELL Technologies).

The study was approved by the Human Research Ethics Committee of Xiangya Hospital, Central South University (AF/SQ 2022090918). The research was conducted according to the World Medical Association Declaration of Helsinki. All the information about the study will be fully explained to the subjects by the researchers. All the participants provided informed consent before sampling.

### Tumorigenesis in nude mice

A total of 10 BALB/c mice (22–28 g, 6–8 weeks) were bought from Hunan Slake Jingda Experimental Animal Co., Ltd. The log-growing MCF cells were centrifuged at room temperature at 500 × g, washed with Hank's balanced salt solution (HBSS) and re-suspended with 50% Matrigel (BD Biosciences). Cell suspension (100 µL) was injected subcutaneously near the adipose pad of the fourth breast at a concentration of 5 × 10^5^ cells/mL [19]. Mice were divided into Control group (PBS) and Exosome group (ASC-exos, 60 μg/100 µL), 5 mice/group. Mice in exosome group was given ASC-exos by the tail vein injection once a day for seven days and then every three days for a total of 28 days [20]. The control group was given equal volume PBS. The tumor was measured weekly using a caliper for the calculation of tumor volume by the modified ellipsoid formula 1/2 × (length × width^2^). This study was approved by the Animal Ethics Committee of Xiangya Hospital, Central South University (AF/SQ 2022090918). All experimental procedures were conducted in accordance with institutional guidelines for the use of experimental animals.

### Cell counting kit-8 (CCK8)

The cells were counted and then inoculated into a 96-well plate (100 µL per well) with a density of 5 × 10^3^ cells/well. After culture adherent, 100 µL CCK8 solution (10:1, NU679, DOJINDO) was added. The cells were incubated and then detected by the Bio-Tek assay (MB-530, HEALES).

### Scratch test

The horizontal lines were drawn by marker behind the 6-hole plate (3516, Corning). After counting, about 5 × 10^5^ cells/well were added. Once the plate was covered with cells, the gun was crossed perpendicular to the line drawn before. The cells were washed with sterile PBS for 3 times. The scratched cells were removed and added serum-free DMEM medium (D5796, Sigma). The scratches were photographed under a microscope (DSZ2000X, Cnmicro) at the 0-h time point, and three different fields of view were captured. After culture for 24 and 48 h, photos were taken again.

### Transwell

The lower part of the chamber (3428, Corning) was supplemented with 500 μL 10% Gibco fetal bovine serum complete medium. Cells (2 × 10^6^ cells/mL, 100 μL) were add to each well and incubated at 37 °C for 48 h. The upper chamber was removed and placed into a new hole with PBS, which was washed 3 times. The upper chamber was fixed with 4% paraformaldehyde for 20 min to obtain the membrane. The membrane was stained with 0.1% crystal violet (G1062, Solarbio) for 5 min and washed. The membrane was placed on the slide to observe cells under an inverted microscope. The chamber was removed and added to 500 μL and soaked with 10% acetic acid for decolorization. At 550 nm, the absorbance value was measured by Bio-Tek assay (MB-530, HEALES).

### Hematoxylin–eosin (HE) staining

Tumor tissue sections were roasted. Sections were dewaxed to water by using xylene and gradient ethanol. Sections were stained with hematoxylin (Abiowell) and eosin (Abiowell), respectively. Sections were observed by a microscope (BA210T, Motic).

### Immunohistochemistry (IHC)

The tumor tissue sections were immersed in 0.01 M citrate buffer (pH 6.0), heated in a microwave until boiling, and then turned off. They were boiled continuously for 20 min. After cooling for 20 min, the slices were taken out and brought to room temperature. After cooling, the slices were washed with 0.01 M PBS (pH = 7.2–7.6) for 3 min × 3 times. The slices were then added to 1% high iodine acid at room temperature for 10 min to inactivate endogenous enzymes. They were washed with PBS for 3 min × 3 times. The slices were incubated with appropriately diluted anti-Ki67 (1:200, ab16667, abcam, UK) overnight at 4 °C. Then, 100 μL of anti-IgG (H + L, SA00013-2, Proteintech, USA) was added to the slices and incubated at 37 °C for 30 min. Next, 50–100 μL of pre-made DAB working solution was added to the slices and incubated at room temperature for 1–5 min, followed by rinsing with distilled water. The slices were counterstained with hematoxylin for 5–10 min, washed with distilled water, and then returned to blue with PBS. The slices were dehydrated with graded ethanol (60–100%) for 5 min at each step. The slices were placed in xylene for 10 min × twice. Finally, the slices were mounted with neutral gum and observed under a microscope (BA210T, Motic).

### Immunofluorescence (IF)

According to the above steps, tumor tissue sections were subjected to antigen retrieval. Afterward, the sections were placed in a sodium borohydride solution at room temperature for 30 min, followed by rinsing with tap water for 5 min. The sections were then immersed in a 75% ethanol solution for 1 min. Subsequently, the sections were placed in a Sudan Black staining solution at room temperature for 15 min, followed by rinsing with tap water for 5 min. The sections were blocked with 15% BSA for 60 min. Tissue sections were dripped with appropriately diluted primary anti-FoxP3 (1:100, PA5-85,236, ThermoFihser, USA) at 4 °C overnight. Then, it was incubated with 100 μL coraLite488 conjugated affinipure goat anti-Rabbit IgG (H + L, SA00013-2, Proteintech, USA). The sections were stained with DAPI working solution at 37 °C for 20 min, followed by PBS rinsing for 5 min, repeated 3 times. The sections were mounted with buffered glycerol and observed under a fluorescence microscope (BA210T, Motic).

### Flow cytometry

The expression of the CD73, CD90, CD44, CD34, and HLA-DR markers in ASC was detected by flow cytometry. In brief, ASC was added and incubated with anti-CD73 (11-0739-42, eBiosciences), anti-CD90 (11-0909-42, eBiosciences), anti-CD44 (11-0441-82, eBiosciences), anti-CD34 (11-0349-42, eBiosciences), and anti-HLA-DR (11-9956-42, eBiosciences) for staining at room temperature for 30 min away from light, respectively. At the same time, the non-dye tube was set. Then, cells were washed by 1 mL PBS and analyzed by flow cytometer (A00-1-1102, Beckman).

To explore the phenotype changes of macrophages, 1 × 10^6^/100 μL cells were added into the centrifuge tube for PBS washing and centrifugation to remove the supernatant. The 0.125 μg anti-CD206 (MA5-23,656, eBiosciences), 0.125 μg anti-CD68 (11-0689-42, eBiosciences), and 0.5 μg anti-CD8 (11-0081-82, eBiosciences) were added to each tube for staining at room temperature for 30 min away from light. Then, cells were washed by 1 mL PBS and analyzed by flow cytometer (A00-1-1102, Beckman). In addition, cells were examined for apoptosis by the annexin V-APC apoptosis detection kit (KGA1022, KeyGEN BioTECH).

To explore the differentiation of CD4^+^T cells, 1 × 10^6^ cells (100 μL) were added to 1.5 mL EP tubes with plus protein transport inhibitors (500 × , 00-4975-93, eBiosciences) for incubation 4 h. Cell precipitate was suspended with Fixation/Permeabilization concentrate (500 μL, 1 × , 00-5123-43, eBiosciences) and fixed at room temperature for 30 min away from light. Permeabilization Buffer (1 mL, 1 ×) was added to the cell precipitation. The cells were suspended, centrifuged, and added 100 μL PBS. Each tube was added with 0.25 μg anti-CD4 (11-0041-82, eBiosciences) and 0.25 μg anti-IL-17A (12-7179-42, eBiosciences) to detect Th17 cells. Each tube was added with 0.25 μg anti-CD4 (11-0041-82, eBiosciences), 0.25 μg anti-CD25 (12-0250-42, eBiosciences), and 1 μg anti-Foxp3 (17-5773-82, eBiosciences) to detect Treg cells. Each tube was added with 0.25 μg anti-CD4 (11-0041-82, eBiosciences) and 0.25 μg anti-IFN-*γ* (12-7311-82, eBiosciences) to detect Th1 cells. The cells were incubated and tested by flow cytometry (A00-1-1102, Beckman).

### Quantitative reverse transcription-PCR (qRT-PCR)

Total RNA was extracted by Trizol (15,596,026, Thermo). Using total mRNA as template, cDNA was synthesized by the mRNA (CW2569, cwbiotech) kit. The target genes (Table 1) were detected by UltraSYBR Mixture (CW2601, cwbiotech) on the RCP instrument (PIKOREAL96, Thermo). β-actin and GAPDH were used as internal parameters. The 2^−ΔΔCt^ algorithm was applied to calculate the expression of target genes.

**Table 1:** Primer sequence

Gene	Primer sequence	Length (bp)
H-PPAR-*γ*	F TGCTCCAGAAAATGACAGACC	194
	R ATTTTCCCTCAGAATAGTGCAAC	
H-HSL	F CACTTAGCCCCTCCACACCCTT	70
	R TCAGCCTCTTCCCCTGCATCCTC C	
H-LPL	F CAATCACAGCAGCAAAACCTT	134
	R GCCAGTCCACCACAATGACA	
H-FABP4	F GGGCCAGGAATTTGACGAAG	184
	R AACTCTCGTGGAAGTGACGC	
H-ADIPOQ	F CATGACCAGGAAACCACGACT	198
	R ACCGATGTCTCCCTTAGGACCA	
H-FoxP3	F CGCCACAACCTGAGTCTGC	81
	R CTCCAGCTCATCCACGGTCCA	
H-IL-17-	F CAGATTACTACAACCGATCCACC	90
	R ACTTTGCCTCCCAGATCACA	
H-IFN-γ	F AGAATGGCTGTGCTGACT	205
	R ATAGCTCTTCGGATACCTC	
H-Arg-1	F TGGACAGACTAGGAATTGGCA	102
	R CCAGTCCGTCAACATCAAAACTA	
H-iNOS	F TCAGCTGTGCCTTCAACCC	199
	R CCGAGGCCAAACACAGCGTA	
H-TNF-*α*	F GAACCCCGAGTGACAAGCCT	120
	R TATCTCTCAGCTCCACGCCAT	
H-CD163	F AAAAGAATCCCGCATTTGGCAGT	184
M-TOX	F GCTCCTCGCACAGAGATCAA	167
	R TTTCTTTTCTCCTGCCCGCT	
M-CD4	F TTCTGGAACTGCACCGTGACC	184
	R TCTCTGCCTTCCACATCAGC	
M-LYZ1	F TCAGGAGGACTAGTGAGCTGT	101
	R CCTGTGGTTATTGGCTGGTACA	
M-CSF3	F GTATAAAGGCCCCCTGGAGCTG	114
	R TGCAGGGCCATTAGCTTCAT	
M-Mettl7b	F CATTACCCACTCTGTCCCCG	85
	R GGCTGCTTTATTGAGTGCCG	
M-Serpinb2	F ATTTCCTGTGTGTCAGCCGC	184
	R CCAGCACCGAGGAGAACTAT	
M-Tbc1d2	F TCCTGTGCCCTGTGAAAACA	112
	R GTGCCCAGATGCTTTAGGGA	
M-GAPDH	F GCGACTTCAACAGCAACTCCC	122
	R CACCCTGTTGCTGTAGCCGTA	
H-actin	F ACCCTGAAGTACCCCATCGAG	224
	R AGCACAGCCTGGATAGCAAC	
H-GAPDH	F ACAGCCTCAAGATCATCAGC	104
	R GGTCATGAGTCCTTCCACGAT	

### Western blot

The proteins in cells and tissues samples were extracted by the radioimmunoprecipitation analysis (RIPA) and lysis buffer. The protein concentration was determined by bicinchoninic acid. Protein samples were isolated by 12% SDS-PAGE. The isolated proteins were transferred to a polyvinylidene fluoride film activated by methanol and sealed with 5% skim milk and dried at room temperature for at least 1 h. The membrane was then incubated with the first antibody overnight at 4 °C, which included anti-CD63 (25682-1-AP, 1:300, Proteintech, USA), anti-CD81 (66866-1-Ig, 1:3000, Proteintech, USA), anti-CD9 (20597-1-AP, 1:600, Proteintech, USA), anti-Tubulin (11224-1-AP, 1:3000, Proteintech, USA), anti-Bax (50599-1-AP, 1:6000, Proteintech, USA), anti-Bcl-2 (12789-1-AP, 1:2000, Proteintech, USA), anti-ERBB2 (#2165, 1:1000, CST, USA), anti-Ki67 (ab16667, 1:1000, abcam, UK), anti-Arg-1 (16001-1-AP, 1:20,000, Proteintech, USA), anti-CD163 (16646-1-AP, 1:600, Proteintech, USA), anti-TNF-*α* (17590-1-AP, 1:600, Proteintech, USA), anti-iNOS (18985-1-AP, 1:600, Proteintech, USA), anti-β-actin(66009-1-Ig, 1:5000, Proteintech, USA), and GAPDH (10494-1-AP, 1:5000, Proteintech, USA). Then, it was incubated with anti-IgG (SA00001-1, 1:5000; SA00001-2, 1:6000, Proteintech, USA). Visualization and imaging analysis were performed by GE Healthcare software (Life Sciences, USA).

### RNAseq

RNA extraction and library construction were performed in tumor tissues. Illumina PE150 was applied for on-machine sequencing to collect the raw data. Fastp (https://github.com/OpenGene/fastp) was used for quality control to obtain the clean reads. HISAT2 was used to compare the filtered clean data with the reference genome sequence to obtain the reference genome. The stringtie software was used to reconstruct the transcript, assemble the exact transcript results, and count the expression of each gene or transcript. DESeq2 or edgeR were used to analyze the difference in expression level with or without biological duplication. Lastly, the Gene Ontology (GO, http://www.geneontology.org/) and KEGG (https://www.kegg.jp/) enrichment analysis was employed to identify pathways exhibiting significant enrichment in differentially expressed genes as compared to the entire reference transcriptome.

### Data statistics and analysis

Graphpad Prism 8.0 statistical software was used for statistical analysis of data in this study. The measurement data were expressed as mean ± standard deviation. First, the data are tested for normality and homogeneity of variance. The test conformed to normal distribution and homogeneity of variance. Unpaired t-test was used between groups. One-way ANOVA analysis or analysis of variance of repeated measurement data were used for comparison among multiple groups. Tukey's was used for the post test. *P* < 0.05 means the difference was statistically significant.

## Results

### Culture and identification of ASC and ASC-exos

**Fig. 1:**
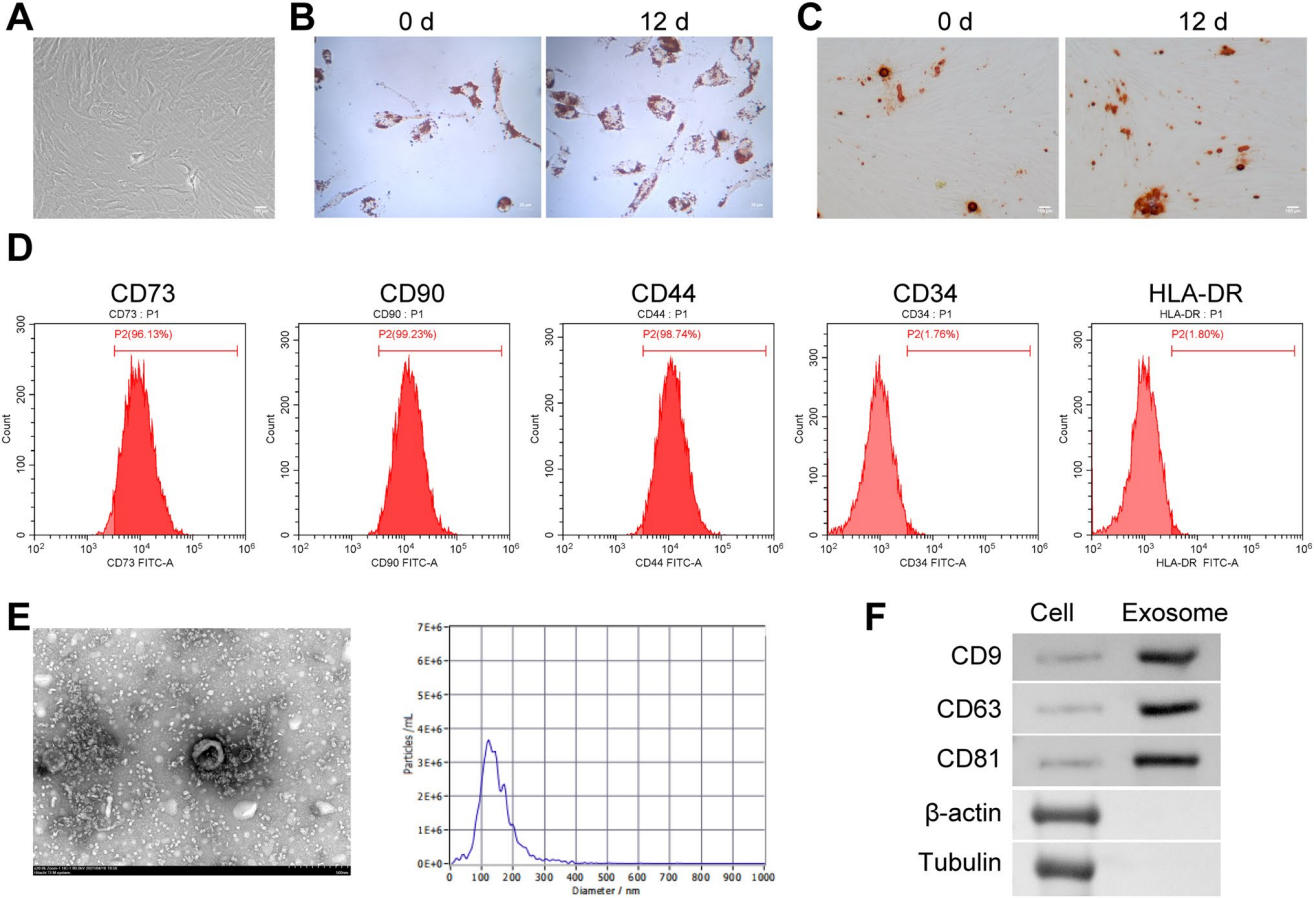
Culture and identification of ASC and ASC-exos. **A** Cell morphology observation of ASC. **B**, **C** Oil red O staining and alizarin red staining was applied to observe the lipogenic and osteogenic differentiation ASC. **D** The expression of the CD73, CD90, CD44, CD34, and HLA-DR markers in ASC was detected by flow cytometry. **E** Electron microscope was applied to observe exosomes. **F** The expressions of CD9, CD63, CD81, β-actin and Tubulin were detected by western blot. *P < 0.05 versus 0 d

As the incubation time increases, ASC gradually becomes homogeneous, forming a monolayer of adherent cells, and displays typical fibroblast-like morphology (Fig. 1A). The third generation of ASCs is collected for pluripotency identification. After 3 weeks of incubation in adipogenic differentiation medium, most cells exhibit characteristics of adipocytes stained with oil red O (Fig. 1B). Similarly, after incubation in osteogenic differentiation medium, most cells show differentiation into osteoblasts stained with alizarin red (Fig. 1C). In addition, flow cytometry characterization reveals that ASCs are strongly positive for CD73 (96.13%), CD90 (99.23%), and CD44 (98.74%) but negative for CD34 (1.74%) and HLA-DR (1.80%) (Fig. 1D). Electron microscopy showed that the diameter of exosomes secreted by ASC was about 100–140 nm (Fig. 1E). The CD9, CD63 and CD81 proteins were highly expressed, while β-actin and Tubulin proteins were not expressed in exosomes (Fig. 1F), which proved that we successfully isolated ASC-exos. The above results proved that we have successfully obtained human ASC and ASC-exos.

### ASC-exos influenced CD4^+^T cell differentiation

**Fig. 2:**
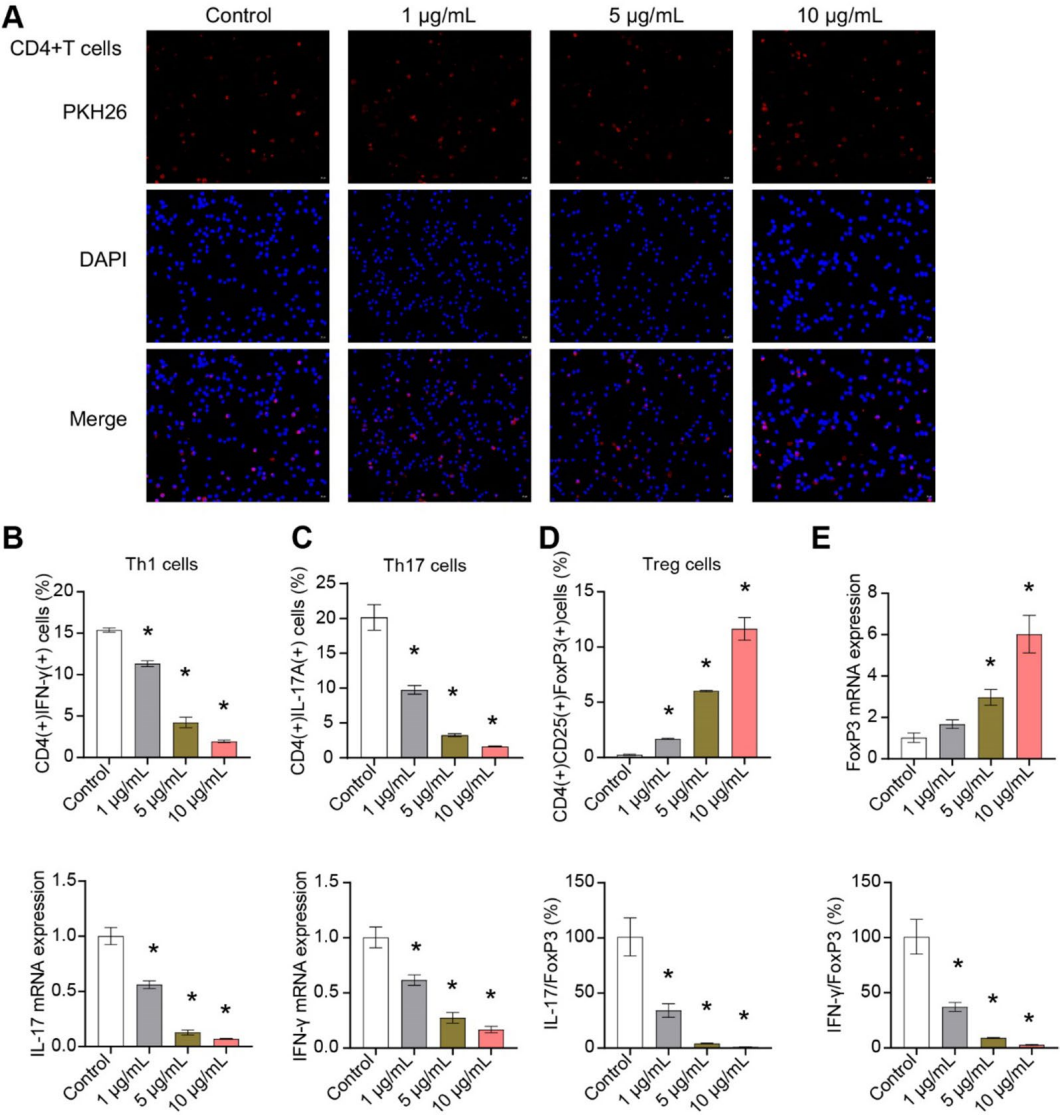
ASC-exos affected the Treg/Th17 cell differentiation in CD4^+^T cells. **A** Cellular localization of PKH26 was determined by exosomes uptake test. **B**–**D** Flow cytometry was used to detect the proportion of Th1, Th17 and Treg cells. **E** The expressions of IFN-*γ*, IL-17 and FoxP3 genes were detected by qRT-PCR. *P < 0.05 versus Control

The PKH26 expression was localized in CD4^+^T cells, indicating that CD4^+^T cells were able to take up ASC-exos (Fig. 2A). ASC-exos inhibited Th1 and Th17 differentiation and promoted Treg differentiation of CD4^+^T cells in a dose-dependent manner (Fig. 2B-D, Supplementary Fig. 1). In addition, ASC-exos inhibited the IFN-*γ* and IL17 expression and promoted the FoxP3 expression in CD4^+^T cells (Fig. 2E). ASC-exos downregulated the mRNA ratio of IFN-*γ*/FoxP3 and IL17/FoxP3 in CD4^+^T cells (Fig. 2E). These results demonstrated that ASC-exos influenced the differentiation of Treg/Th17 cells in CD4^+^T cells.

### ASC-exos influenced macrophage differentiation from CD14 + monocytes

**Fig. 3:**
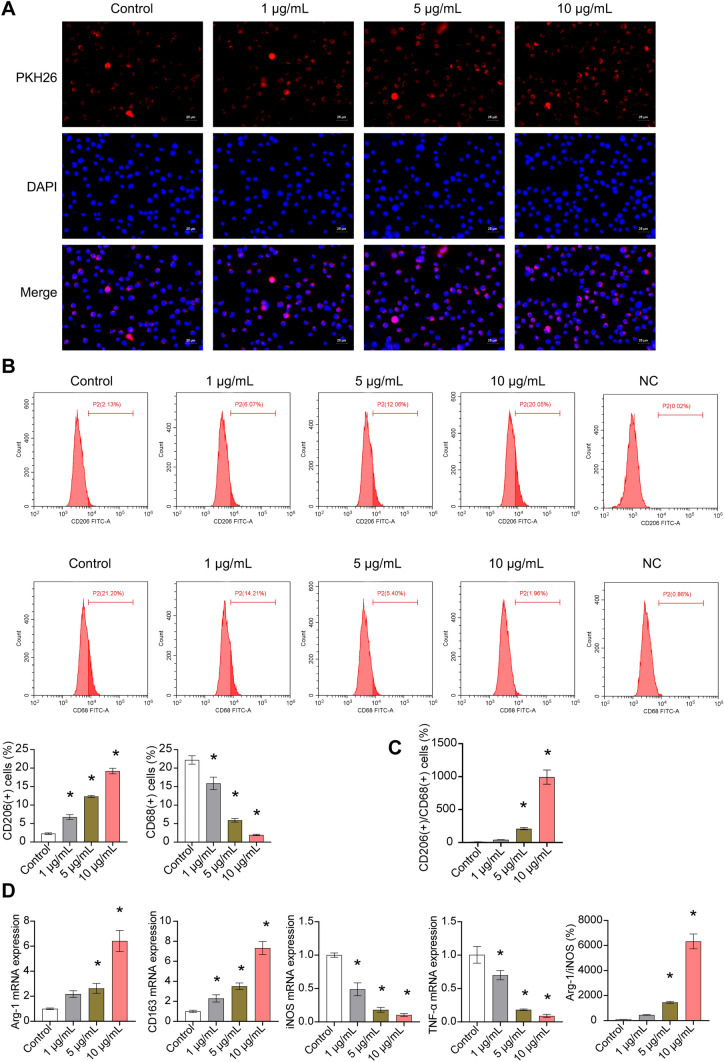
ASC-exos influenced macrophage differentiation. **A** Cellular localization of PKH26 was determined by exosomes uptake test. **B**–**C** The proportion of M1 and M2 cells were determined by flow cytometry. **D** The expressions of Arg-1, CD163, TNF-*α* and iNOS were detected by qRT-PCR. *P < 0.05 versus Control

IF staining showed that macrophages could ingest ASC-exos (Fig. 3A). ASC-exos inhibited M1 differentiation and promoted M2 differentiation of macrophages in a dose-dependent manner (Fig. 3B). ASC-exos increased the CD206( +)CD86( +) macrophages (Fig. 3C). In addition, ASC-exos promoted the Arg-1 and CD163 expression and inhibited the TNF-*α* and iNOS expression in macrophages (Fig. 3E). ASC-exos upregulated the mRNA ratio of Arg-1/iNOS in macrophages (Fig. 3D). These results demonstrated that ASC-exos promoted M2-type differentiation of macrophages.

### ASC-exos affected the characterization of MCF-7 cells

**Fig. 4:**
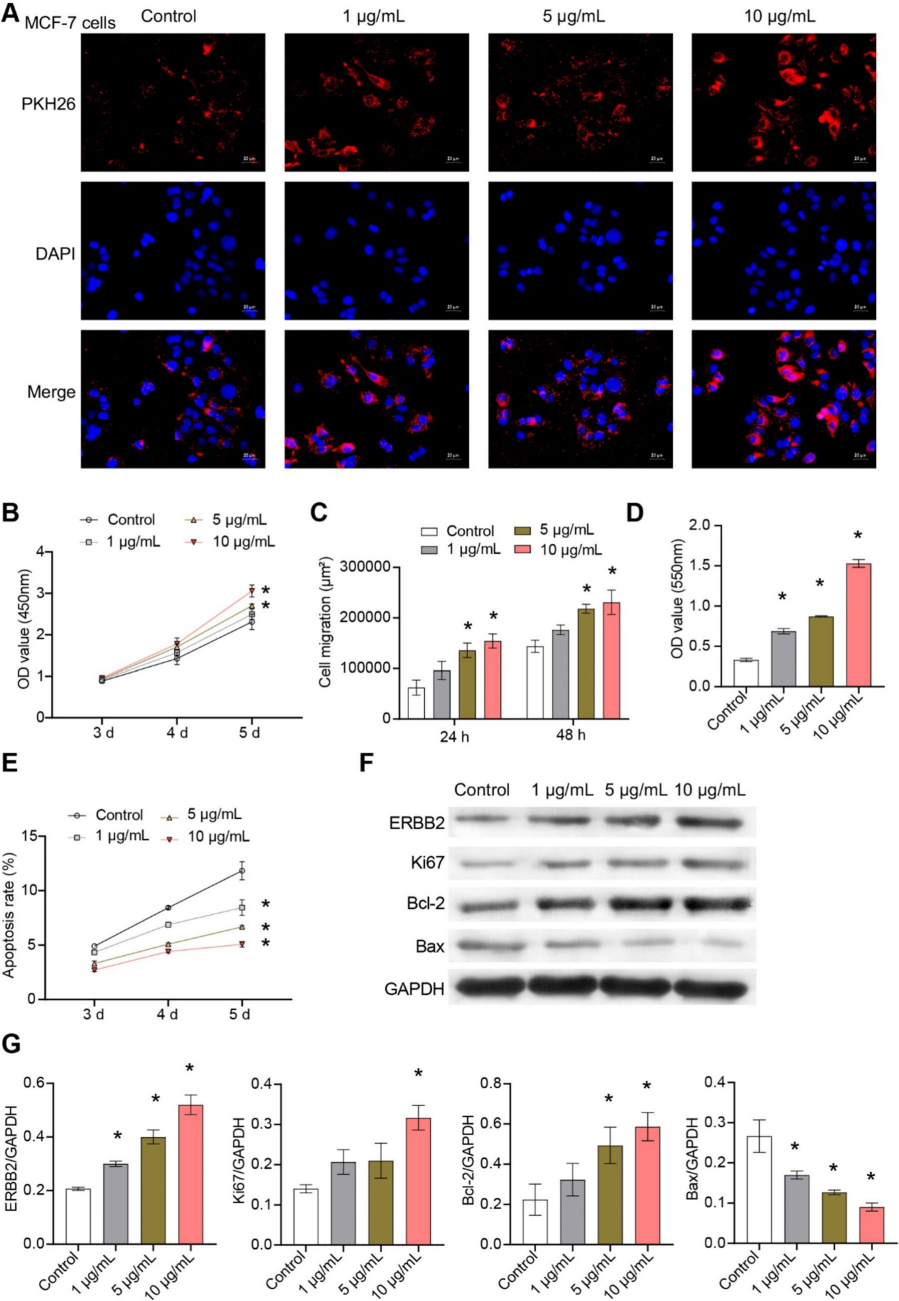
ASC-exos affected the basic characterization of MCF-7 cells. **A** Cellular localization of PKH26 was determined by exosomes uptake test. **B** Cell proliferation was detected by CCK8. **C** Cell migration was detected by scratch assay. **D** Transwell was used to detect cell invasion. **E** Apoptosis was detected by flow cytometry. **F**–**G** The expressions of Bcl-2, Bax, ERBB2 and Ki67 were detected by western blot. *P < 0.05 versus Control

IF showed that MCF-7 cells could take up exosomes from ASC (Fig. 4A). ASC-exos promoted the proliferation, migration, and invasion of MCF-7 cells in a dose-dependent manner (Fig. 4B–D, Supplementary Fig. 2A,B). In addition, ASC-exos inhibited apoptosis of MCF-7 cells (Fig. 4E, Supplementary Fig. 2C). ASC-exos promoted the expression of Bcl-2, ERBB2, Ki67 and inhibited the expression of Bax in MCF-7 cells (Fig. 4F–G). These results demonstrated that ASC-exos promoted the basic characterization of MCF-7 cells.

### ASC-exos promoted the formation of breast cancer in nude mice

**Fig. 5:**
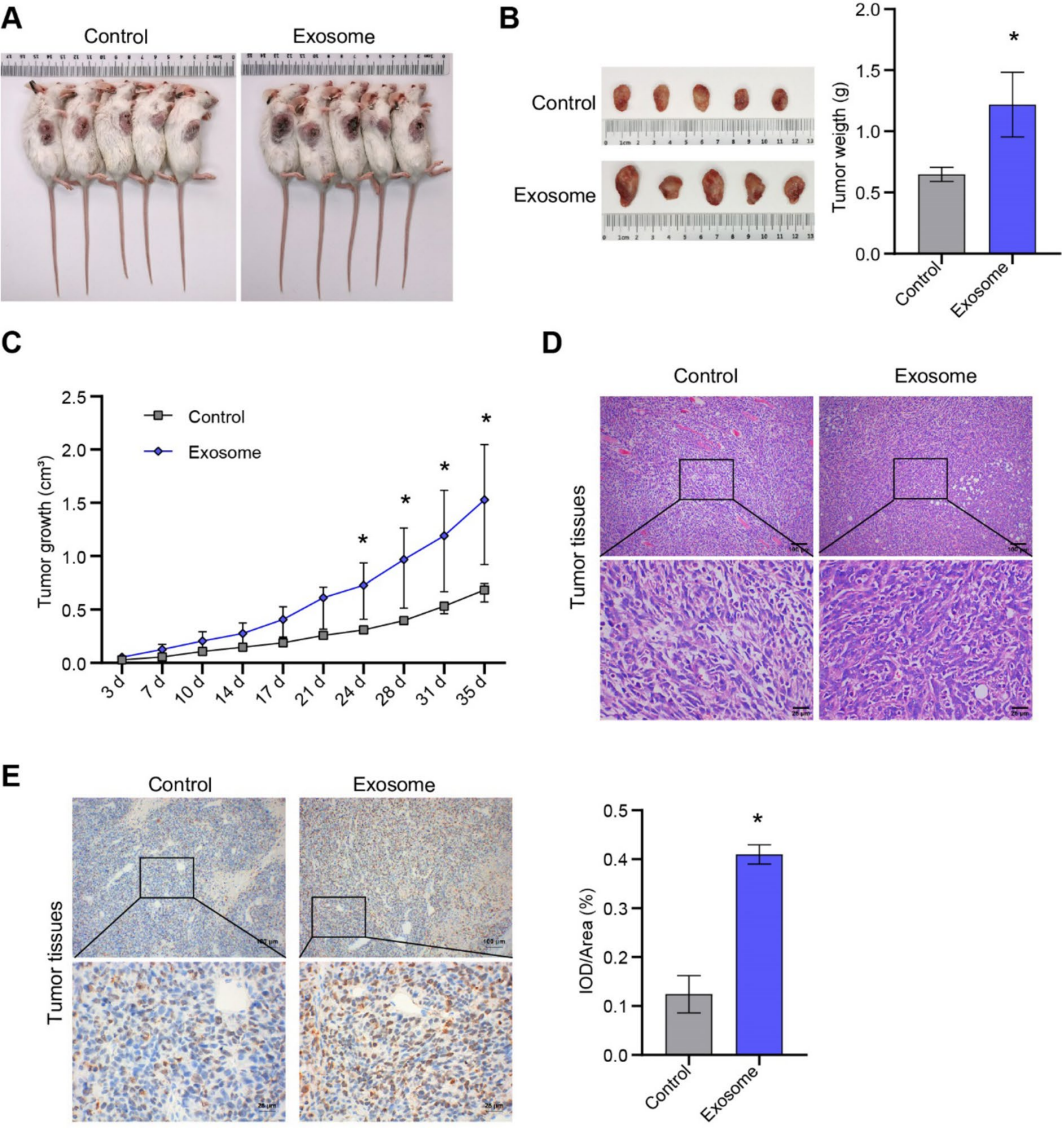
ASC-exos promoted breast cancer formation. **A** Tumor formation was observed in nude mice. **B** The observation and weight analysis of tumor tissues. **C** Tumor volume cure. **D** HE staining was applied to observe the pathological changes of tumor tissue. **E** The expression of Ki67 was observed by IHC. *P < 0.05 versus Control

Nude mouse tumorigenesis showed that ASC-exos promoted tumorigenesis of breast cancer cells (Fig. 5A, B). ASC-exos significantly increased the tumorigenic volume and weight of MCF-7 cells (Fig. 5B, C). HE staining showed that compared to the control group, there were more red blood cells and tumor cells in the exosome group (Fig. 5D). IHC analysis found that ASC-exos increased the Ki67 expression in tumor tissues (Fig. 5E). All the results proved that ASC-exos promoted the development of breast cancer tumors.

### ASC-exos influenced tumor microenvironment immunity of breast cancer

**Fig. 6:**
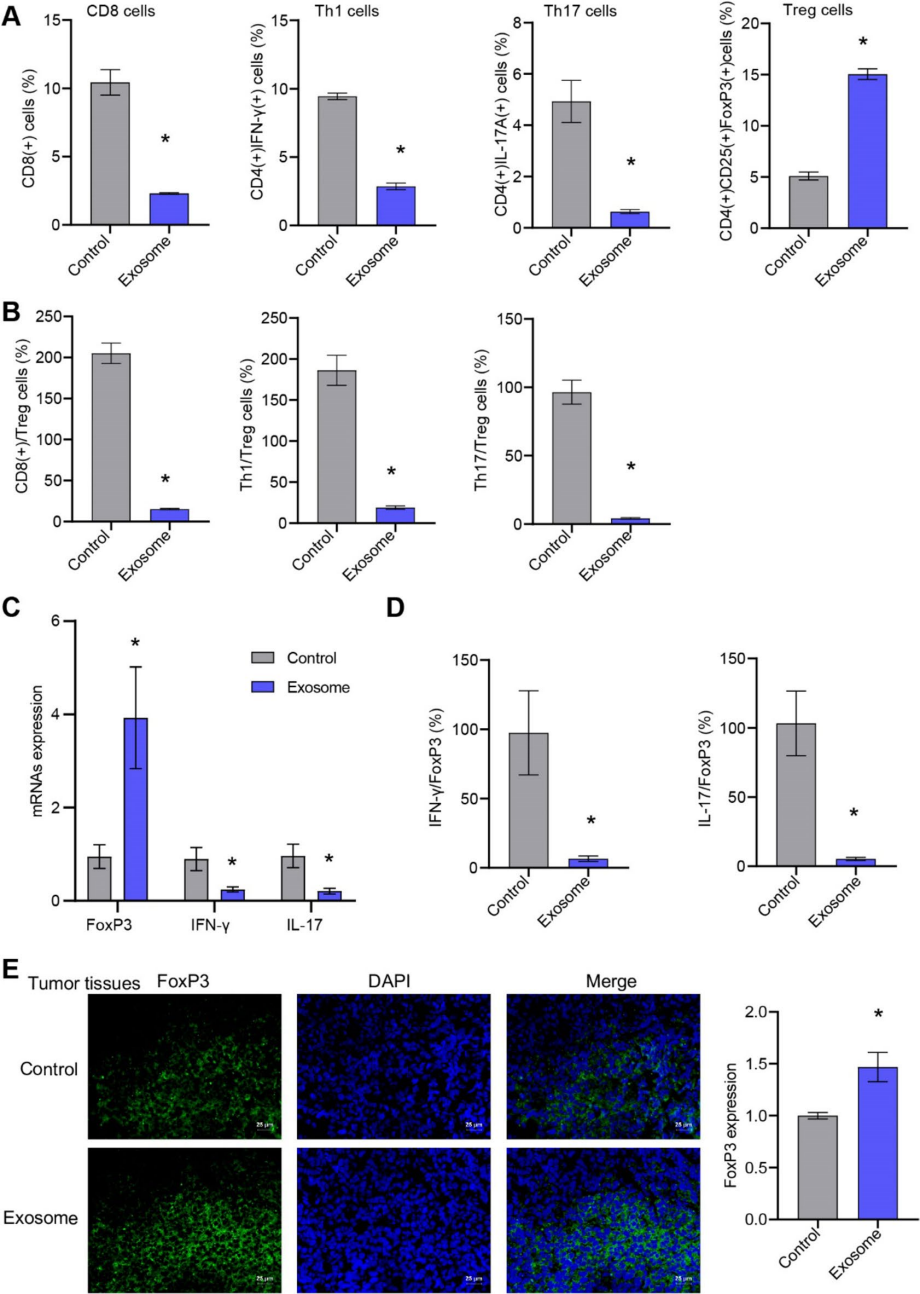
ASC-exos influenced tumor microenvironment immunity of breast cancer. **A**, **B** The proportion of CD8, Th1, Th17, and Treg cells in tumor tissue were tested by flow cytometry. **C**, **D** The expression of FxoP3, IFN-*γ* and IL-17 in tumor tissues was detected by qRT-PCR. **E** The expression of FxoP3 was detected by IF (25 μm). *P < 0.05 versus Control

ASC-exos inhibited the differentiation of CD8, Th1 and Th17 cells, and promoted the differentiation of Treg cells in tumor tissues (Fig. 6A, B, Supplementary Fig. 3). ASC-exos promoted the FxoP3 expression and inhibited the IFN-*γ* and IL-17 expression in tumor tissues (Fig. 6C, D). IF further confirmed that ASC-exos promoted the FxoP3 expression in tumor tissue (Fig. 6E). ASC-exos influenced the tumor microenvironment immunity of breast cancer.

### ASC-exos influenced tumor macrophage differentiation in breast cancer

**Fig. 7:**
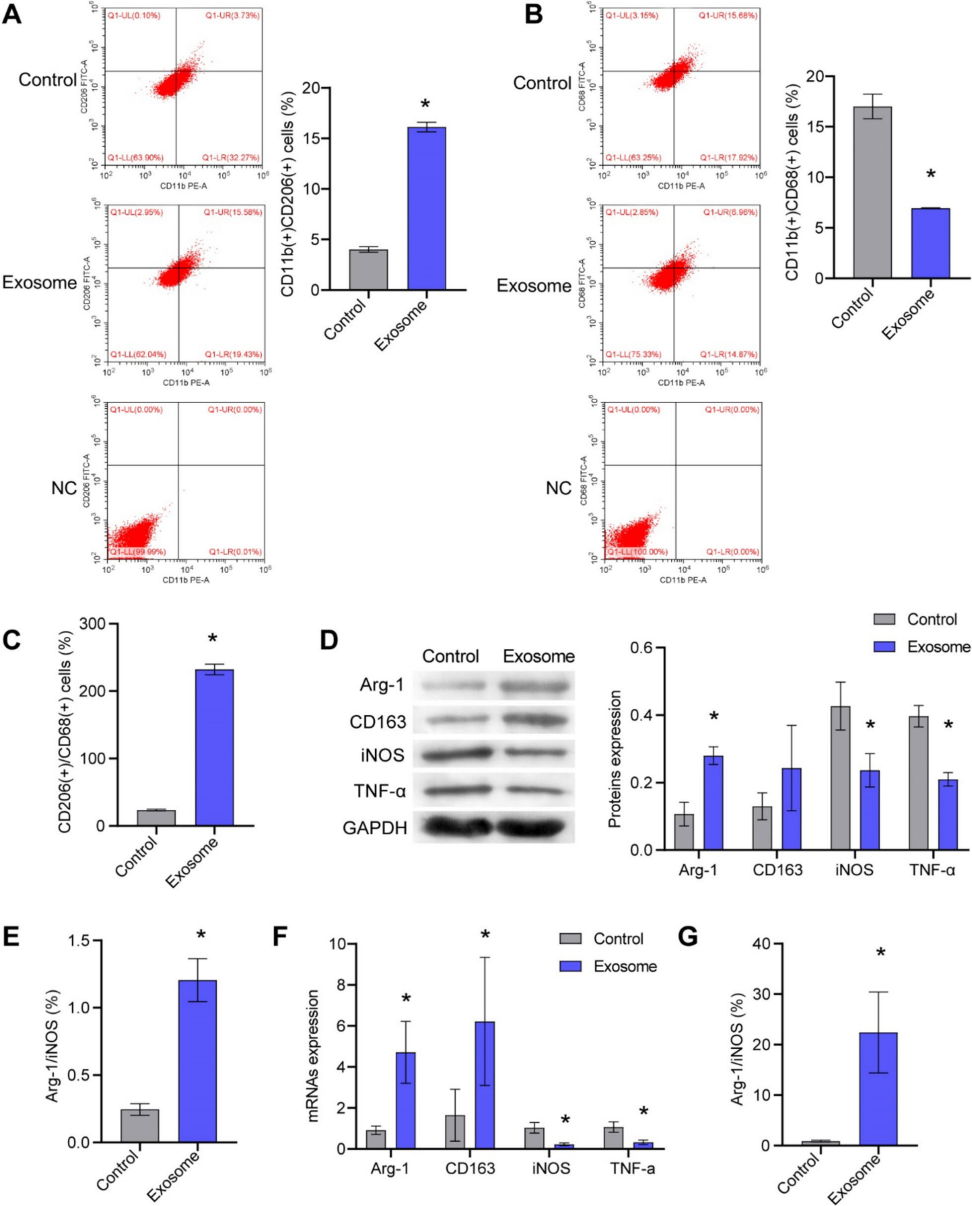
ASC-exos influenced tumor macrophage differentiation in breast cancer. **A**–**C** The proportion of M1 and M2 macrophages in breast cancer was analyzed by flow cytometry. **D**–**G** The expressions of Arg-1, CD163, TNF-*α* and iNOS were tested by qRT-PCR and western blot. *P < 0.05 versus Control

ASC-exos promoted M2-polarization and inhibited M1-polarization of macrophages in breast cancer tumor tissue, thereby worsening the tumor (Fig. 7A, B). ASC-exos increased the CD206(+)CD86(+) macrophages (Fig. 7C). In addition, ASC-exos promoted the Arg-1 and CD163 expression and inhibited the TNF-*α* expression and iNOS (Fig. 7D–G). ASC-exos upregulated the ratio of Arg-1/iNOS expression in macrophages (Fig. 7E and G). These results demonstrated that ASC-exos promoted the polarization of M2-type macrophages in breast cancer and worsen the tumor.

### ASC-exos influenced T cell function in breast cancer

**Fig. 8:**
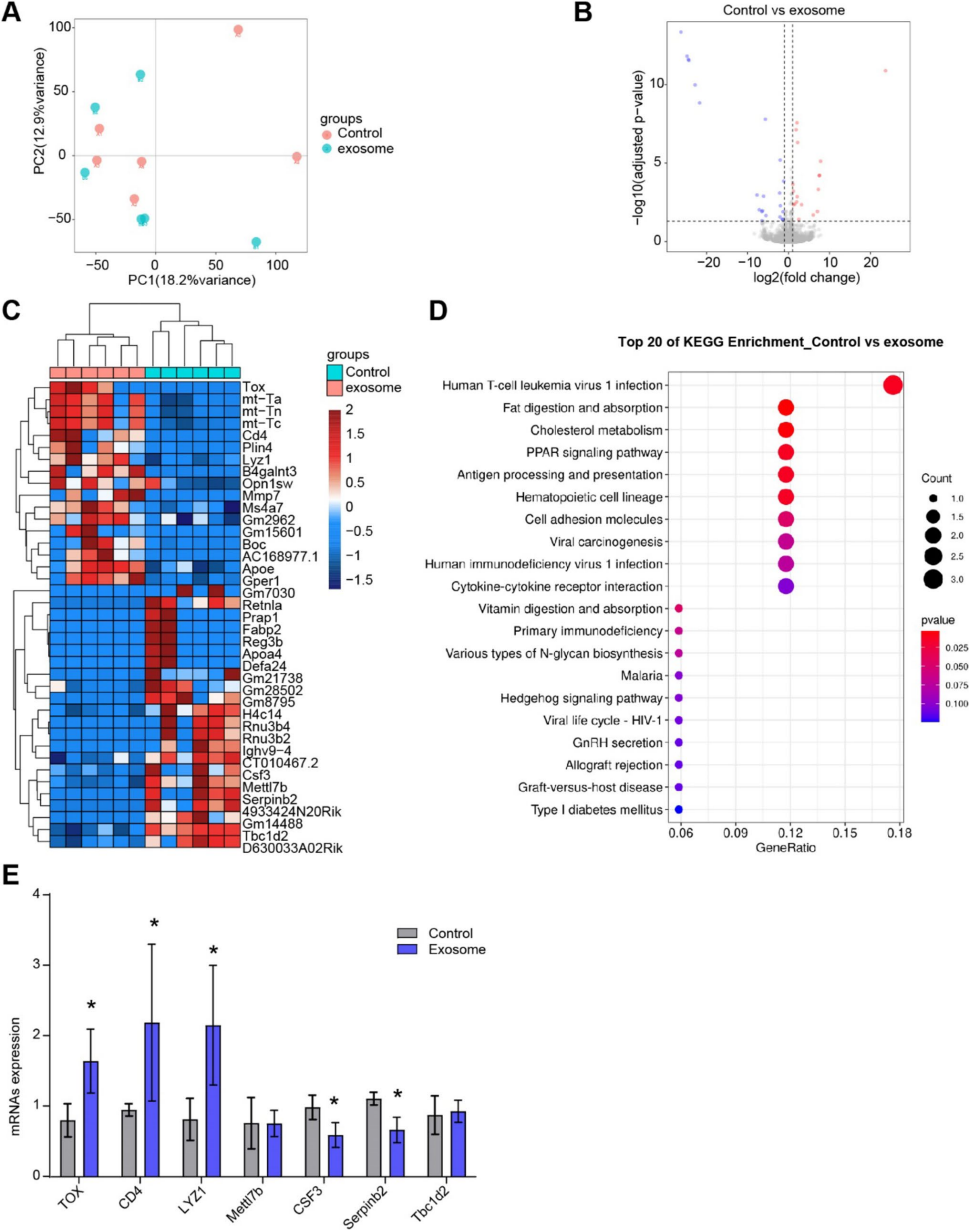
ASC-exos influenced T cell function in breast cancer tumor tissues. **A** PCA analysis. **B** volcano plot. **C** Heatmap showed the gene abundance. **D** KEGG was applied to predict the function of differential genes. **E** qRT-PCR was applied to verify the differential genes expression in breast cancer tissues. *P < 0.05 versus Control

We used RNAseq to analyze the changes of RNA expression profile and function in breast cancer tumor tissues before and after exosome intervention. PCA analysis showed that the samples from control group and exosome group showed discrete distribution, with crossover and deviation (Fig. 8A). Volcano map proved that 17 genes were higher and 22 genes were lower under the ASC-exos treatment (Fig. 8B). Heatmap showed variations in the abundance of 39 differential genes (Fig. 8C). KEGG functional enrichment analysis showed that the Human T-cell leukemia virus 1 infection pathway was significantly enriched in exosome group (Fig. 8D). The TOX, CD4, and LYZ1 genes were higher, and the CSF3 and Serpinb2 genes were lower in exosome group, which was consistent with sequencing results (Fig. 8E). These results proved that ASC-exos affected T cell function in breast cancer tumor tissues.

## Discussion

The tumor microenvironment plays an important role in inducing cancer cells to enter dormancy and participate in reversible epithelial-mesenchymal transition (EMT) to an aggressive phenotype that leads to cancer-related death in patients [21]. Direct cell-to-cell contact between cancer cells and ASC via exosomes vesicle exchange caused breast cancer cells to exhibit changes toward a more malignant phenotype [22, 23]. All these were associated with higher rates of metastasis and a worsening prognosis [22]. Exosomes secreted by adipose tissue-derived MSCs could induce apoptosis and inhibit metastasis of breast cancer cells by repairing miR-145 [24]. Adipose-derived MSC-exosomes could deliver miR-1236 to increase the sensitivity of breast cancer cells to DDP by participating in SLC9A1 downregulation and Wnt/β-catenin inactivation [25]. Adipose-derived MSC-exosomes were successfully isolated and internalized by MDA-MB-231 cells, and miR-381 mimics were effectively delivered to MDA-MB-231 cells by the MSC-exosomes [26]. Our in vitro and in vivo studies confirmed that ASC-exos promoted the migration, proliferation, and invasion of MCF-7 cells, inhibited apoptosis in a dose-dependent manner. These studies confirmed that ASC-exos promoted malignant characterization of breast cancer cells.

In addition to its direct effect on tumor cells, obesity also systematically provided a prerequisite for future metastasis of the tumor microenvironment by promoting the formation of pro-inflammatory niches [27]. Exosomes of adipose-derived MSC ameliorated experimental colitis by inhibiting Th1/Th17 cells and macrophages and reducing the pro-inflammatory cytokines level [28]. ASC-exos promoted angiogenesis and cancer cell migration and have neuroprotective and nerve regeneration properties [29]. The delivery of miR-424-5p targeting PD-L1 by EVs derived from adipose tissue MSC promoted proinflammatory effects [30]. Adipose tissue-derived MSC-exosomes with miR-10a promoted Th17 and Tregs responses while reduced Th1 responses [31]. Our studies confirmed that ASC-exos inhibited Th1 and Th17 differentiation of CD4^+^T cells, promoted Treg differentiation, and inhibited M1/M2 differentiation of macrophages, which were partly consistent with the above studies.

ASC have been shown to favor tumor progression in several experimental cancer models, playing a central role in regulating tumor aggressions and metastatic potential through a variety of mechanisms, such as paracrine release of exosomes containing cancer-promoting molecules and induction of EMT [32]. Tumor-derived exosomes induced myofibroblasts phenotype and function in ASC through a SMAD-mediated signaling pathway, thereby promoting tumor cell progression and malignancy [33]. MSC-exosomes promoted the migration of breast cancer cell line MCF7 and were associated with WNT signaling pathway activation [34]. Small extracellular vesicles from immune cells and other donor cells can be engineered to provide better targeting and biological effects [35]. All these studies were consistent with our study, which confirmed that ASC-exos influenced the tumor microenvironment immunity of breast cancer to promote the malignant characterization of breast cancer, provided new evidence for ASC-exos.

However, conflicting evidence has emerged regarding the safety of ASC applications in recent years [36]. Extracellular vesicles regulate a variety of cancer-related processes by transmitting homologous and heterologous cell communication cues [37]. The intervention of exosomes from osteoblast ASC in cancer stem cells could significantly reduce the expression of ATP-binding box (ABC) transporters, breast cancer gene family (BCRA1 and BCRA2), ErbB gene family and other drug-resistant genes, helping to overcome the treatment resistance [38]. In addition, drug delivery via exosome could reduce the cell viability of MCF7 cell and without any significant cytotoxicity to normal L929 cell, which has great potential for clinical application [39]. ASC conditioned media could induce selective stress by increasing cell proliferation, resulting in a more aggressive phenotype in MCF-7 and MDA-MB-231 cells [40]. Our results confirmed that ASC-exos promoted the TOX, CD4, and LYZ1 expression and inhibited the Mettl7b and Serpinb2 expression in breast cancer tumors, which were significantly enriched in the Human T-cell leukemia virus 1 infection pathway. Therefore, ASC exosomes promote the development of breast cancer by regulating the expression of RNA expression profiles in breast cancer tissues.

This study focuses on the analysis of the effects of ASC-derived exosomes on the malignant characteristics and tumor formation of breast cancer cells. In addition, ASC-exosomes are rich in proteins, lipids, and nucleic acids, serving as crucial mediators in key biological processes in cells and tissues through intracellular signaling mechanisms [41]. Given the limitations of proteomics technology and experimental funding, we took a different approach and used RNA-seq to explore the effects of ASC-derived exosomes intervention on the RNA expression profile in tumor tissues. But the specific contents of ASC-derived exosomes were not clearly analyzed. This limitation of our study needs to be further explored in future research. Further exploration of the potential functions of proteins, lipids, or nucleic acids in ASC-derived exosomes may provide new directions for our next study, but some potential technical issues still need to be addressed. However, our study does provide initial evidence that ASC-derived exosomes promote the malignant characteristics of breast cancer and play a role in regulating the tumor microenvironment immune response, which highlights the significance of this research.

In conclusion, ASC-exos promoted breast cancer characterization and tumor microenvironment immunosuppression by regulating macrophage typing and T cell differentiation-related functional gene expression.

### Supplementary Information

Below is the link to the electronic supplementary material.

Supplementary file1 (JPG 289 KB)

Supplementary file2 (JPG 1071 KB)

Supplementary file3 (JPG 2419 KB)

Supplementary file4 (JPG 746 KB)

## Data Availability

The data that support the findings of this study are available from the corresponding author upon reasonable request.

## Abbreviations

ASC: Adipose tissue-derived stem cells
ASC-exos: ASC-exosomes
HE: Hematoxylin–eosin
HBSS: Hank's balanced salt solution
IF: Immunofluorescence
RIPA: Radioimmunoprecipitation analysis
