# Supplementary figures and images for: Adipose-derived stem cell exosomes promote tumor characterization and immunosuppressive microenvironment in breast cancer
Source: Cancer Immunol Immunother. 2024 Jan 31;73(2):39. doi: 10.1007/s00262-023-03584-3 (PMC10830720; doi:10.1007/s00262-023-03584-3)

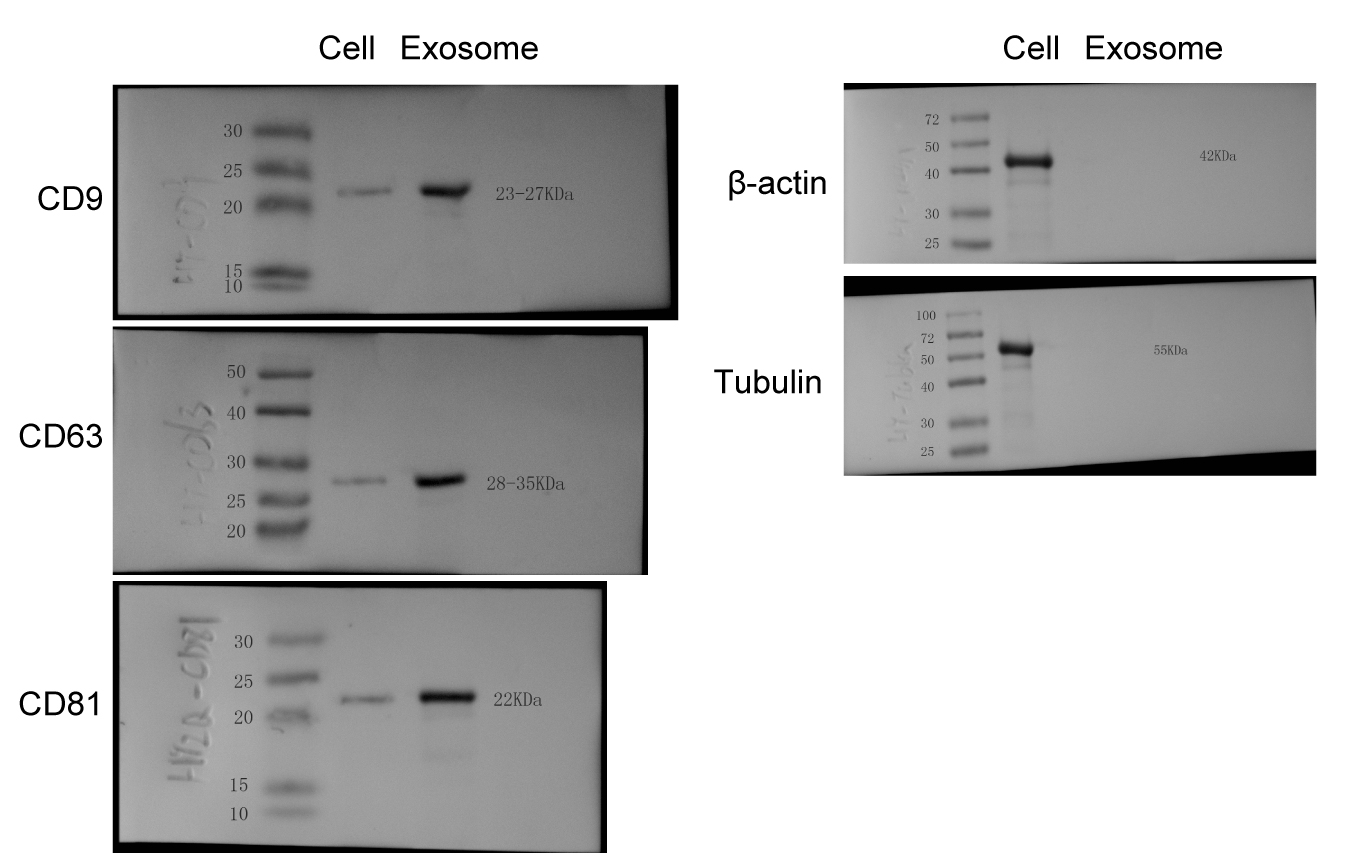

Supplement: Supplementary file 1 — Supplementary file1 (JPG 289 KB) [file 262_2023_3584_MOESM1_ESM.jpg]

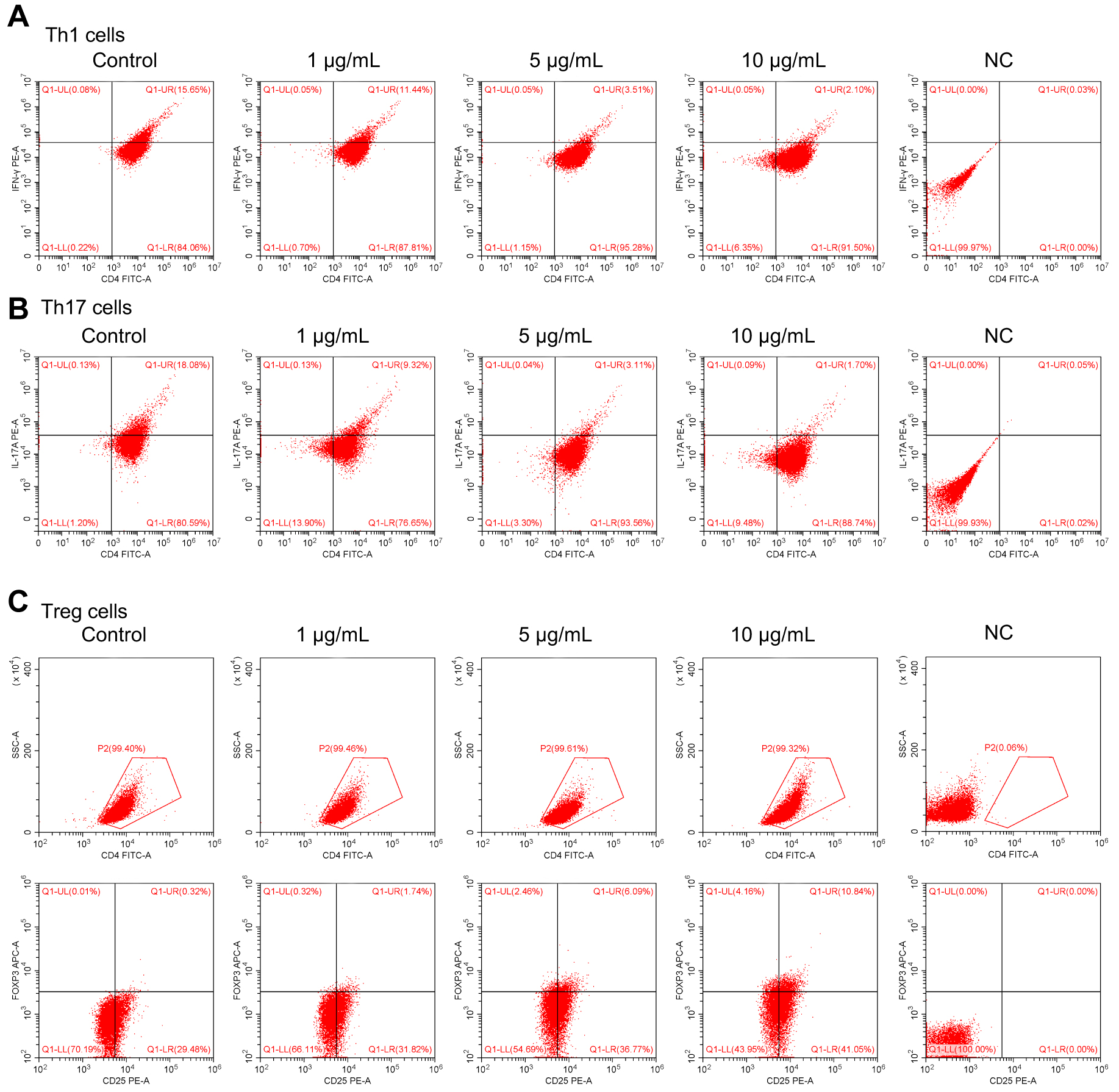

Supplement: Supplementary file 2 — Supplementary file2 (JPG 1071 KB) [file 262_2023_3584_MOESM2_ESM.jpg]

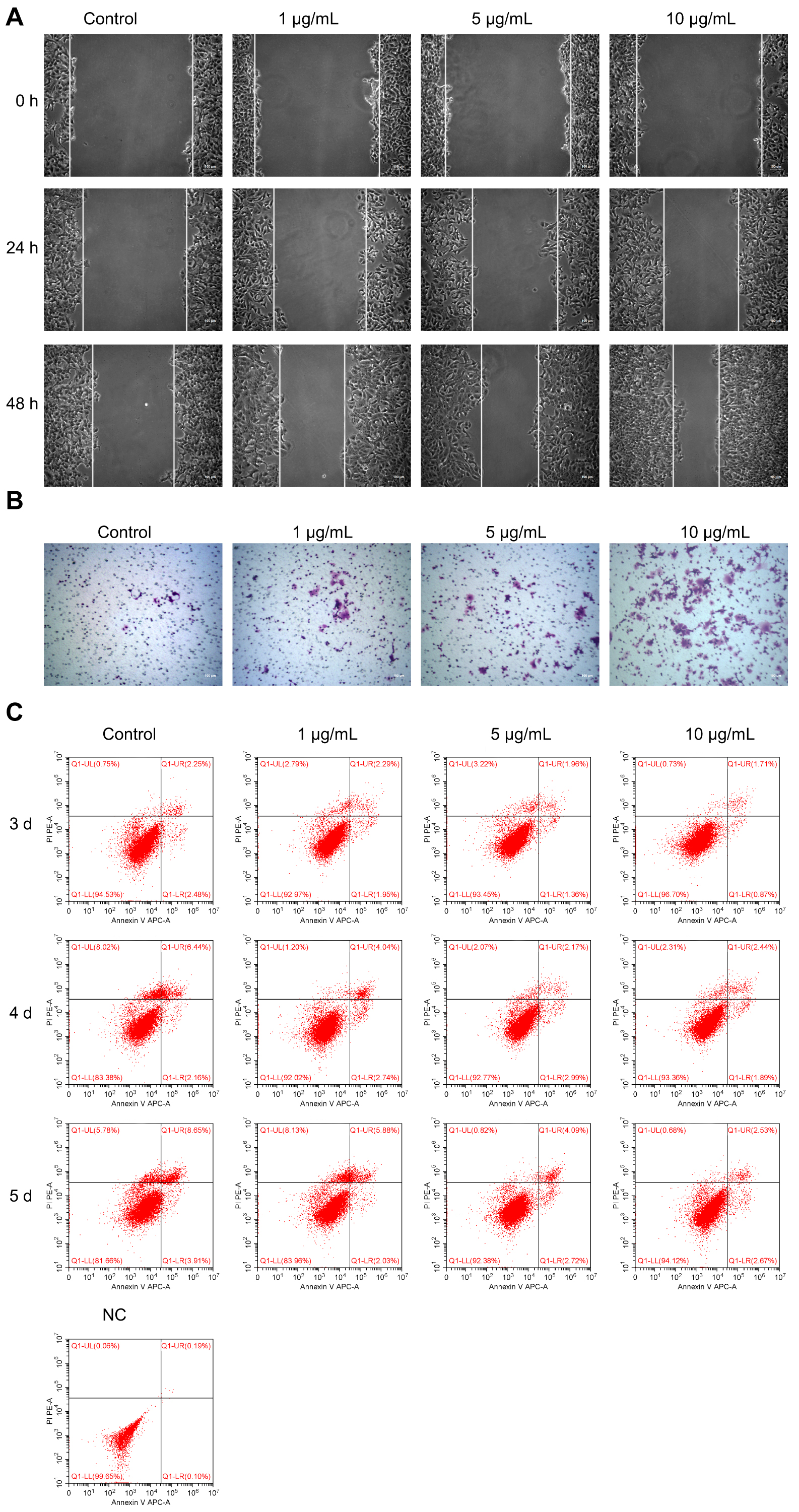

Supplement: Supplementary file 3 — Supplementary file3 (JPG 2419 KB) [file 262_2023_3584_MOESM3_ESM.jpg]

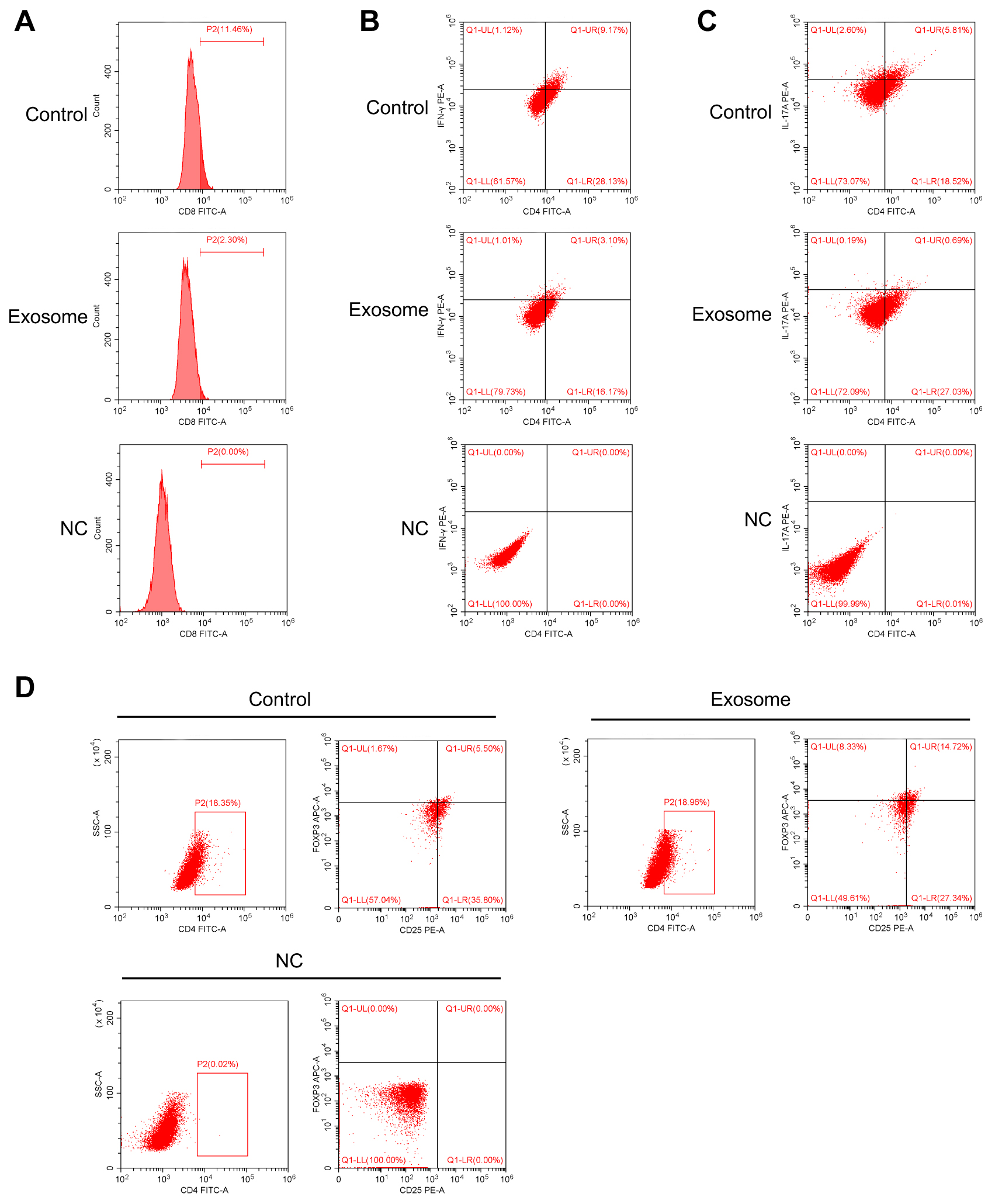

Supplement: Supplementary file 4 — Supplementary file4 (JPG 746 KB) [file 262_2023_3584_MOESM4_ESM.jpg]
